# Supplementary material for: Is quality maternal healthcare all about successful childbirth? Views of mothers in the Wa Municipality, Ghana
Source: PLoS One. 2021 Sep 15;16(9):e0257401. doi: 10.1371/journal.pone.0257401 (PMC8443026; doi:10.1371/journal.pone.0257401)
Supplement: S1 Data — (DOCX) [file pone.0257401.s001.docx]

**Transcription of interviews held**

**Section II: Service users’ perception about what constitutes quality maternal healthcare**

It is about weighing of children, giving us drugs to ensure that we deliver well. They are good, they welcome us with smiles, the nurses actually assist us to deliver successfully.

**Section III: Appraisal of quality of maternal healthcare provided in WA municipality**

The place we deliver has just one bed, when a woman is already on labour on the one bed the subsequent ones have to sleep on the floor with a piece of cloth. The midwife always travels on Fridays to the Wa municipality, when it happens like that we have to travel to Wa to the regional hospital. For good delivery, the midwife has to be around all the times.

The maternal wards are not spacious enough, they don’t have all the items, equipment and tools for healthcare services, they don’t have water and blood for patients that required them. When a woman delivers and need that, the nurses have to go to a nearby village/CHIP to request for. The midwife and nurses are human, when they see you coming they run towards you, they say good things to us. Sometimes they are hungry yet still working and taking care of us. We are suffering and they are also trying their best.

The major difficult we encounter is the referral, when we are given referral to Wa it becomes difficult for us to get there. we have to hire a car. I was pregnant and water was running out of my genital, I thought I was due to give birth, so I want to the health center, they examined me, inserted their figures into my vaginal and realised that the child was not closer, yet the water was flowing, so they had to refer me to the regional hospital. When I got there, they requested for my referral letter and I gave it to them and they assisted me through the delivery. The nurse that assisted me during the delivery had that humanity.

When you get to the regional hospital, they will tell you to buy this and that, but here the nurses don’t ask us for anything. We don’t pay for deliveries and it is satisfactory, the few items we are always able to send to the CHIP compound during the deliveries they accept. It is always annoying when we are given referral. When the nurses here detect that you have low blood also they refer you to Wa hospital.

Whenever, we are due delivery, they always request for pads that they will use in collecting the blood during the delivery, we mostly suffer to get that to buy in this village even when you have the money.

**Section IV: Challenges service users face in seeking quality healthcare during delivery**

Because we are rural dwellers we are not always comfortable when we are referred to the regional hospital and face a lot of people we don’t know. But because the CHIP compound does not have all the equipment, they have not all the equipment for deliveries, and not always able to attend to pregnant women whose babies are not laying well in their stomachs and need to go through caesarian. Such women are often referred. If everything is available in their CHIP compound it would have been good to us the rural dwellers who fear the nurses in the regional hospital because we are not familiar with them.

When just have only one midwife who conduct deliveries here. Also, when they request for a scan in the early pregnancy times, we normally had to walk to Bamahu to do the scan. We sometimes have to leave the result and come home or sleep over there just to wait for the results and bring it back to the nurses. if we had gotten a laboratory diagnosis center here we would have been happy.

RECOMMENDATIONS

We mostly are not always able to buy the rubber pads the nurses normally request for during the delivery, we will be happy if the nurses could buy them and put them in the health center so that anytime we go and need them we can buy from them.

Two or more nurses in addition to the current ones will be adequate for us, particularly those who can conduct deliveries. We also hope that at the end of every week, at least one can remain at the health center.

**Section II: Service users’ perception about what constitutes quality maternal healthcare**

The kind of medication to prevent complication, the mode of reception of patients by nurses, some try to take your hand bag. Non-harassing of pregnant women, by the nurses. The nurses not asking of unnecessary questions from the nurses. Good encouragement from the nurses may encourage you the give birth again. Quality healthcare is one without operations. But the way the nurses used to treat us those days is not the same as today. Sometimes you will go to the health center, the nurses will tell you that it is weekend and they have to go home and prepare food.

**Section III: Appraisal of quality of maternal healthcare provided in WA municipality**

Giving birth at the health center has no quality. Sometimes we still go to the CHIP and give birth without the supervision of the health workers. We are always afraid to go to Wa hospital because of this. The cost of assessing delivery services is much higher in the regional hospital than the CHIP compounds within the community. We don’t have enough beds here, we have just two beds one for other sick people and the other for delivery of women. Two women cannot give birth at the same time, if you get there at the same time then one has to sleep on the floor to deliver and the other on the bed. If you really not patient you will fight. A week ago, a woman came around to the health center and ended up giving birth in the urinal, one woman was referred back to her room a home to go wait where she ended up giving birth without the supervision of the health workers. The nurses that had the human face has been transferred.

**Section IV: Challenges service users face in seeking quality healthcare during delivery**

The absenteeism of nurses affects us a lot. The accuses the nurses give normally give are not reasonable. They will tell you they are going cook whilst you are on labour suffering.

The frequent referral of nurses from the facility also affect our access to healthcare. Because of that we are often referred to the Wa hospital and when it happens like that we have to hire a car and pay Ghc 30.00 which is not easy for our poor husbands. When you are expected to be motivated by the nurses and you don’t get that it discourages us.

When you carry your ward to the health center you will just start insulting you, they will not wait patiently for you to tell them what is even wrong with the child before. They always say it is because of the NHIS that is why we come to the health center frequently.

The Community Health Nurses used to assist the midwife when she is not around but I don’t know why she does not assist us any longer. In the absence of the midwife they always refer us.

Language barrier is also another thing, sometimes you meet some of the nurses who cannot speak our language and that challenges our diagnosis. They cannot ask you of your health situation.

**RECOMMENDATIONS**

We think if we have three midwives in addition to the already existing ones it would have helped us a lot. some pregnant women don’t require referrals but because of the limited number of midwives they just refer us unnecessarily.

We can also discuss this with the nurses, the need for them to motivate pregnant women through their speeches and words. There were days the nurses used to sit with us and speak with us the way forward but these days we don’t get that any-longer. Here the nurses end up insulting you the client, it is always like they are annoyed already waiting for you. The used to change duties from one nurse to other at the weekends, but now all of them leave in every week-ends. The previous in-charged used to motivate us with soaps.

**Section II: Service users’ perception about what constitutes quality maternal healthcare**

R2:First of all when you are pregnant and come to the chips compound , the lab test they do to check that you and the baby stay health is quality maternal healthcare to my understanding

R4:I also think that we pregnant women know very well when we come to deliver that the nurses cannot pull out the baby unless the time is due but we always expect that once we carry ourselves here to deliver we are in the hands of the nurse and therefore have no control over our own selves. And so we expect the nurses to understand us and not maltreat you and so to me the quality is when one comes to deliver and fall in the hands of good nurses who will treat you well and try to share your pain with you.

R1:Well I will say that the quality here is better than noon because even if we come to deliver we do not get a place to hung our lets and also nothing to hold and push for the baby to come out and also the nurse for now are good to us we not know if they will change in future because the wicked nurses who were here are all gone.

**Section III: Appraisal of quality of maternal healthcare provided in WA municipality**

***R6:As we have mentioned earlier we do not have a ward at all so there is no space to talk about a portion of the place were consultancy is done has been divided with curtains for delivery and there is just one bed there which is even not a delivery bed and so when two or more come here to deliver only one can be catered for and also there is no place to hung one’s legs or even to hold to assist us to push during delivery***

*R1:The personnel are not enough at all there are only four nurses here with a very large population to take care of and so they have to divide the duties especially weighing days for some to be here and others on the field. and because of this no even weighing of our babies is done strategically area by area.*

*R2:The tools and equipment are not much . The is only midwife here who is sometimes supported by a male nurse here to conduct deliveries.*

*R6:well the last time I came to deliver I was well taken care of so I will say they are reliable and responsive to me and also there was that empathy for me the midwife really took very good care of me.*

*R7: For the last time I care to deliver that was when the nurses who have been transferred were still around when my caretakers brought me she was inside but told someone to come and tell us that she was not around and it was midnight my husband had to struggle t get a car and rush me to Wa regional hospital and at down another woman came to deliver and that was when she confessed that she was actually around but the people worry her so much and that is why she did that to me.*

*R2:yes for this current nurses they are timely to call to duty. And on the part of communication during delivery it depends on the midwives at the time of deliver because when you meet good nurses they talk to you nicely and encourage you to push but when you meet wicked ones they insult you and they you that you are pretending .*

*no non of us have been referred before.*

*yeah we are okay with the overall services of this facility. the processes are that when you come, the nurses check your cards and ensure that your month and date is really due and then they deliver you.*

*R3: YES the processes are satisfactory and affordable and the things that we have to buy before we come to* deliver *is also fine since it is used to clean the mother and baby.*

*R8: yes delivery service is accessible to us and also if we are to rate we will give them 80% because it is just a small clinic and the services they render is due to the resources available to them. Unlike the big hospital that has more .*

**Section IV: Challenges service users face in seeking quality healthcare during delivery**

There are some green drugs that the clinic does not have to give us when we are pregnant and we go to buy which is a challenge

Also, we do not have a maternity ward here.

There is no toilet facility here and so when you come to deliver and at a point you attend you attend to the nature’s call in the bush you can born there since there is no toilet in the hospital

Also there is no water here so we the pregnant women fetch water to fill the poly tank so that when any of us come to deliver the nurses will get water to do their work

RECOMMENDATIONS

A new big clinic has been built and it contains everything but it is not opened for use so we want then to open for us to save us the cost of travelling to Wa to deliver.

Also the green drugs should be provided to us

Also we need water in the clinic here

Also we will be happy if the nurses would educate us during weighing to educate us on the injections they give to our babies and also when there is a new outbreak somewhere they should pre educate us I experience that when I was in one community but or this community the nurses just inject the baby and go away

**Section II: Service users’ perception about what constitutes quality maternal healthcare**

They take good care of us, they don’t worry us. If you expected to bring some items during the delivery, they normally informal us before the delivery period. They don’t insult us, they take care of us as we expect. For me when I delivered, they gave me tea to drink and the time they gave me key soap for the washing of children things.

**Section III: Appraisal of quality of maternal healthcare provided in WA municipality**

The bed is too small for deliveries, it does not also have the metallic push that assist us during delivery to push easily. We also have a mid-husband that assist us in deliveries. The nurses are timely and responsive, even when the person travels she/he insists that you come to the CHIP and they do come and attend to us. By the time you reach the health facility, she/he is already at the facility.

**Section IV: Challenges service users face in seeking quality healthcare during delivery**

We fill shy of the mid-husband who assist us in the deliveries, we would have been very comfortable if he was a female. We are not comfortable anytime we are due delivery.

I witnessed a situation at the regional hospital where a pregnant woman said she does not want to be assisted by a mid-husband to deliver, so they didn’t say anything but just ignored and attended to me. For me the males are even better than the females’ midwives, they are able to handle pregnant women better than that of the females during deliveries.

Lack of access to decent transport challenges our willingness for referral to the regional hospital. It is always a problem and fearful whenever we are referred to the Wa hospital because of the caesarian and the means of transport issue. The last time they referred me I had to get a motor king. Whilst on the way I felt the sun rises on my stomach because it is an open means of transport. On my way I delivered and still had to manage to the health center in Wa for the check-up.

The nurses are not also many to assist us, the few around are helpful for deliveries and they are good so far, we don’t have travel to Wa often.

My brother wife delivered the child in the WASH room and the nurses beat her at the health center. she didn’t know that she needed to take a rubber container and urinate inside, and she went to the WASH room and ended up giving birth there.

As villagers when we are not lucky and we meet a wicked nurse at the health center we suffer a lot. They will treat you like you are not a human being. But if you are new mother the nurses here usually refer you to the regional hospital to avoid complications.

**RECOMMENDATIONS**

The provision of moto king would have facilitated the movement of referred patients from here to the Wa hospital. Also, the provision additional beds with metallic pushed to ease delivery among pregnant women. Other facilities such as scanning machines will also help us a lot. It sometimes takes a month to get lab results just because we have to travel to the regional hospital.

**Section II: Service users’ perception about what constitutes quality maternal healthcare**

**R3** Quality maternal healthcare is the care we get when we go to hospital during pregnancy and during delivery unlike when we used to born at home

**R2** Quality maternal health care is the good care given to us by nurses when we go to deliver.

**Section III: Appraisal of quality of maternal healthcare provided in WA municipality**

1.R3 well for the general evaluating I will say it not bad at all but sometimes it depends on the nurses/midwives on duty at the time of visit. There are times one goes to meet nurses who are very good to you and get all the time to interact well with you but others do not.

R4 Also we used to think that the hospital was a scary place to go so when we are pregnant or in labour we just stay at home but now because of the quality maternal healthcare given to us by the nurses we always prefer to go there and deliver safely and come home with our babies in good health.

2.R1 for me, I will say is good because the last time i visited there the nurses were very good and had so much time for me by interacting with me

R5the health facility has good healthcare services even though there are times you meet some midwifes who insults and even punish you make a small mistake. when I visited the health facility last two months to deliver two nurses nearly fought because of me because one was not happy the way the other was handling a woman in labour badly so I think their services are good.

The health facility is very spacious maternity ward enough for many women to deliver. There are enough beds at the facility due to the renovation they did and none of us have ever gone to sleep on the floor there and there are always a good number of nurses and midwives in the ward to take care of us.

The tools and equipment at the health facility are enough and functioning very well and as soon as one is due to deliver everything s there to take care of the process. There are a lot of midwives who do deliveries themselves, they are very reliable and quick to respond to clients.

R1 the midwives are very timely to call because when a nurse is responsible to deliver you, she sits by you throughout especially at night to constantly check and take care of you. Even when the nurses go to sit at their table and you call to them they are quick to come to your aid.

R6 the nurses are very empathetic but there are times that the attitude of we the women in labour forces the nurse to shout at you to enable you to be serious to push. Because when at a point during labour the woman tries to complicate things that could lead to death of the baby, they lose the empathy and discipline you.

R2 Also, when the pain of labour starts and you immediately run to the hospital thinking the nurses will perform magic to save you meanwhile your time is not due to deliver and due to the pain you are going through you over worry the nurses, they sometimes lose empathy for you.

R4 The nurses communicates with you right from the time you come to deliver, for instance they ask you to know the number of children you have, where you delivered each of them either at home or hospital. During the delivery process they tell you what to do and what not to do to make the process easier and for the baby to come out safely.

3.R1 yes I have been referred to the WA municipal hospital from the Kpongu clinic here . the midwife here told me that my baby was not positioned well so I had to go to the bigger facility. from my experience there was really no much difference in the experience between the clinic here and there.

R3 I went there no because I was referred but just to also to experience how delivery was done there but the difference was not much even though they have more nurses there.

4.We are all satisfied with the overall maternal healthcare services in the facility.

R1. Immediately you get there they collect your health insurance card and maternity book and ask your care taker to go for your folder.

R3 they then check you BP and also use some equipment to touch your stomach to see if there is enough water for baby to turn

R4 After all these processes if you are not still due they give you a bed to rest and constantly come to check you to see how the baby is turning.

R6 when your time is due you are delivered after which the mother is given two injection on her thighs immediately for good health. they then ask you if u are dizzy or not and also check the baby’s health . The next day they come to check the mother and baby and inject the baby and drops some medicine into his mouth to prevent some disease .

we are very comfortable with the process because it helps to improve good health .

R1 I was asked to pay just ₵6 Ghana cedi’s when I went to deliver to me it affordable because I was able to afford it . I also bought my Dettol and others

R3 I also paid ₵3 Ghana cedi’s

R5i paid ₵7 Ghana cedi’s. we think it is good though we buy the Dettol, rubber gloves and others even though costly but they are used to clean the place and also used to lean we and our babies to look lean and fresh

Delivery services required is accessible because the distance is not far from us and even if you are referred you a easily get there in no time

R1 I will rate the facility 100percent because the facility has been renovated and also there are separate places for those who deliver themselves and those that are operated on because the operated ones need more care e.g being helped to breastfeed babies and their wounds being cleaned and to me I think it a very good thing.

**Section IV: Challenges service users face in seeking quality healthcare during delivery**

R3 When you get to the health facility to deliver and you do not have money to buy the Dettol, parraZon, layer, rubber, pad and gloves it becomes a problem because you will not be discharged if u do not pay after the facility have used their products to deliver you. and this forces us to deliver at home if you do not have money.

R6 the major problem to me is if you buy all the above mentioned items if not all is okay the place is well renovated.

R2 I did not encounter any challenge

R4 my only challenge is that I had to buy all my things almost ₵100 Ghana cedi’s and it a worry to most of we the mothers.

**RECOMMENDATIONS**

IF they could reduce the things we need to buy during delivery it will help a lot. Because it makes you think a lot when the time is due.

**Section II: Service users’ perception about what constitutes quality maternal healthcare**

I gave birth to my first child there, when you go the nurses will chart with you to make you comfortable, even after delivery if you get a tear and they are suturing you, they will be talking to you till they finish and give you a bed to sleep and when is time for you to go home they will allow you to go.

**Section III: Appraisal of quality of maternal healthcare provided in WA municipality**

Am not very satisfy with the delivery process. a case where u go the hospital to delivery, these “*small small*”, will sit and expect you who is in labour to spread yr cloth and rubber and be waiting for the baby to coming. All they do is sit with their phone koroko! Ko! Ko!.... while you are suffering….sometimes when they prescribe drug for you to buy, they ask you who is in labour to get to the pharmacist to get the drug especially if you don’t have a guidance .

There is no privacy in the regional hospital, if u really know where the women are delivered, it is expose to public view. Sometime kurooo u don’t get bed to sleep on. They have to flow you

For the nurses at our facility, they are very good to us, they response to all our delivery needs right from conception to labour. They educate us, organizes pregnancies classes for us. All these help boost our delivery. We have a midwife who conduct delivery. The problem is that, they are not enough. For instance, when they are two or three people in labour at the same time. There will be serious problem. The same thing have to do with our bed. Is only one we have here? Even the one, is not as conformable as what we have at our regional hospital’s. With the one at regional hospital, they have places where u fix your legs. Unlike our own, u can even fall in the place. All these are creating some kind of discomfort in our delivery process (**teacher**)

**Referral**

…….Actually when we went to the facility that faithful Saturday, the facility was locked. My mother-in-law and I have to rash me to the hospital (Wa Regional hospital). If we relented at the point and decided to go home and been attended by a TBA, I would have lost my son because what they brought out of my sons toe after I gave birth was not something small, I was just imaging how all these tins would have happen delivering after the nurses at the facility failed us.

I could also die in the process of going to the regional hospital to deliver. Because the road from Charia connecting Wa is rough and full of pot holes …..the kind of balancing this motor kind ridder have to take me through could have killed or aid me loss my son… hmmmmmm but do I really have a choice.

**Payment of delivery services**

Usually when you deliver at the health facility you don’t pay anything. But when a mother fail to buy iteam (Detol, pampers, rubber etc) the always ask very pregnant women to bring, u are detained at the hospital till your people come to pay you off.

…….for that one dear we don’t but the kind of things the nurse ask as to pay at the delivery ward is more that payment….typical was when I delivered my daughter I was injected some medicine, after delivery I was injected twice, but they took the antenatal book and later give you so it was there I was told to pay 6ghanacedis for the injection and that one is 3ghanacedis it was not the man that injected me that too the money and I also told they bought pad to add to what I brought and used on me which was also 10ghanacedis. With this I confidently say we don’t pay…

**Challenges**

The bed for delivery are not enough, Midwives who conduct the delivery are few, as such, the few available are always stressed and lastly Poor staff interpersonal relationships to us and our guidance

**Recommendation**

Increase our nurses and Increase delivery beds and make it a bit comfortable

**Section II: Service users’ perception about what constitutes quality maternal healthcare**

The checking that he nurses give when you are pregnant and giving you drugs to keep you and the baby healthy and also the laboratory test they do for you immediately you get pregnant and another before you deliver to ensue that mother and baby are healthy and free from diseases and finally the care given by the nurses to ensure mother is delivered safely and baby is also healthy to me is quality maternal healthcare.

**Section III: Appraisal of quality of maternal healthcare provided in WA municipality**

**1.**To me the services rendered was very good unlike delivering at home, the nurses take good care of you and take out all the dirty blood clots so when you get home you do not experience any afterbirth problems. Also when we visit during pregnancy we are given drugs to take that make mother and baby strong and healthy, they also reposition the baby well if it is not in good shape and if it is not breathing they tell you . so to me it a good thing.

2.Well it is better than noon because the ward is not really spacious and so if three pregnant women get sick or are in labour and meet here at the same time, one is referred to WA. there are only two beds in the maternity ward and to me I think it is not enough. The personnel are not really much they are about five (5) but they are doing well, they try all their best to take care of us. There were enough tools and equipment which were used as at the time I came here last year(2017) but for now I cannot really tell. There is only one midwife here and she does deliveries with the help of a community health nurse(female) here.

Yes the midwife is very serious , reliable and responsive to clients, immediately one gets here, you are attended to immediately . And anytime a woman in labour gets here and meet her absence, the community health nurse is always around to take care of you till she arrives because she stays in Wa and comes here to work.

They are very empathetic with us, they try to encourage you during delivery. Yes they are very timely to duty call even when you get here at midnight they come and attend to you.

Yes the community with us during delivery. they tell you o be patient and push and also advice you not to cry and that everything will be okay.

3. No I have never been referred to any health facility.

4.Yes am satisfied with the overall maternal healthcare services received here. because if you listen to the advice of the nurses and do what is required of you, you will see go results at the end unlike women who do not listen to them sometimes end with complications.

When you get here to deliver, the nurses take you maternity book and health insurance card and also check you things including Dettol, rubber ,parazon, geisha soaps racks and pad.

they then deliver you after which they clean up the mother and baby and also ensure that you the mother is not having excess bleeding.

The process is very good because my first I had at home and this one I had in the hospital is different so the hospital process is good . it is very affordable because health insurance covers all the only difference is the few things that you buy. It is very accessible to me because my house is just behind the clinic. If am to rate will rate them the best or 100percent because I have neve come here and the nurses handled me carelessly so am happy.

**Section IV: Challenges service users face in seeking quality healthcare during delivery**

1. the ward is not spacious enough and so when three pregnant women get sick and come here at the same time only two(2) will get space the third have to go to Wa.

There are times that we get here and we are given few drugs and we are asked to buy the rest.

2.The challenge that I faced the last time I came was the sleeping place to deliver because I foresaw that if a third person had come there won’t space for her to deliver

3. I will recommend that the government should add at least another building in this facility for us to get space and also add more beds for more women because even the nearby chip ones refer women here sometimes.

THANK YOU.

**Section II: Service users’ perception about what constitutes quality maternal healthcare**

Quality health care is care given to me to be healthy after delivery. When I was pregnant and went to the facility, the nurse lay me on the bed and measured my stomach and asked my health situation ad gave me some drugs to take. After birth, they give drugs to the child and when it is done they insist that you bring the child to the facility when she/he is not feeling well. During pregnancy we go for lab test and start the antenatal care process.

**Section III: Appraisal of quality of maternal healthcare provided in WA municipality**

I delivered my first born in the room and the two children in the facility. The services they provide to us are very good. Anytime am sick and get to the facility, they take good care of me. The room is spacious but the delivery bed is just too small and only one bed. When two women are on labour, the midwife has to be in haste to finish with the first one and continue with the other person. We have enough nurses (3) but I don’t know the number of the nurses in the facility that can perform deliveries. The nurses have equipment but I don’t know whether they have all of them or not.

The nurses conduct deliveries, they are always available and ready to conduct delivery at all times. They have humanity in them; when I was pregnant and due labour walking towards the facility, when they saw me coming they ran towards me and took my bag and the items. I was very happy. I entered the facility and they performed the delivery.

I am very satisfied with the maternal healthcare services in the facility. when you are due delivery, they asked you to bring Dettol parasol, soap, rubber and pads. During the delivery, they normally ask you to lay on the bed and they will put hand gloves in their hands and insert their fingers into your vagina to find out whether they child is closer or not. When you cannot push by yourself, they normally provide a machine to help you push out the child. The service is affordable. In this village we don’t spend money or pay money for deliveries. Giving birth is easier and accessible also, we are encouraged more to give birth their services. When we are pregnant there is nothing to fear at all, they will tell you to come around anytime you are pregnant to seek early antenatal care services

**Section IV: Challenges service users face in seeking quality healthcare during delivery**

We normally have to travel all the way to Wa to do lap test, screening particularly in our early days of pregnancy. The beds are not enough for delivery, additional beds will help us. We also need more midwives to perform deliveries.

**RECOMMENDATIONS**

If we get a more spacious facility with additional midwives and beds it would have been helpful to us.

**Section II: Service users’ perception about what constitutes quality maternal healthcare**

The nurses are taking good care of us, when you go to the facility they will first ask you what is wrong with you and depending on the situation, they prescribe drugs for you to go home and take. They normally advise us to come back to the facility when we are not feeling ok after taking the drugs.

**Section III: Appraisal of quality of maternal healthcare provided in WA municipality**

They are generally good, I was on my way coming to the facility but unfortunately delivered on my way. The following day I went to the facility and they injected me, assessed the baby and removed the remaining blood in my body.

Just that the rooms are not spacious enough, with one delivery bed. We have three (3) permanent nurses here, they have equipment and tools for working and they are functioning. They are always available to us anytime we are due labour. They have that human empathy. I was due labour and on my way I unfortunately delivered and went back home. The following morning, I went to the facility and they asked me what happened and I explained to them and apologized for not being able to reach the facility. They accepted and took care of me. I am satisfied with their services so far. The services are also affordable and of good quality.

**Section IV: Challenges service users face in seeking quality healthcare during delivery**

I don’t have any challenge with the healthcare system.

**RECOMMENDATIONS**

If we get a more spacious facility with additional midwives, beds it would have been helpful to us

**Section II: Service users’ perception about what constitutes quality maternal healthcare**

I think quality maternal healthcare is when u go to deliver and meet a god nurse who does not shout at you but have the patience with you and take care you, the care we also require is that you do not met nurses who will maltreat you in the labour room. but on other days you can meet nurses who also teat you badly but you meet others who say good things to you are take care of you till you deliver.

for me when I went to deliver I really had quality care because the nurses I met were running after me and sharing my pains with me and cleaning me up so I think it was quality.

I also think quality maternal healthcare is the monthly retain drugs that they give to us as well us the checkup they do on us to ensure that the baby is moving and breathing and also ensure that the mother is healthy.

**Section III: Appraisal of quality of maternal healthcare provided in WA municipality**

**1**.For me I will say the services of the Dondoli chips is better than the Wa regional hospital because I took my monthly retain drugs here even though I did not deliver here because when you come for the drugs they give you enough attention and do not waste your time unnecessarily

**2**. R1, R2, R4: None of us delivered here so we do not know much concerning the spaciousness of the ward, beds available and personnel.

I took my retain drugs here but I do not have enough information on the personnel here at the Dondoli clinic and also during my monthly visits here they has tools to check me for instance there is a funnel like equipment they use to listen to the breath of the baby but I think they do not have all anyway.

But at the regional hospital there enough tools and equipment that they use t deliveries. Also, there are enough personnel because the last time I went to deliver three midwives were in the delivery room with me including a man aside the other in the ward they were very caring because I had to squeeze the man’s hand because I had o where to hold and push and he really supported me and I just pushed once and the baby was out.

I also think the regional hospital has enough personnel very young and caring nurses in the labour room. if you do not enter the labour room sometimes u fell so fed-up and angry because the elderly women at the table in the ward are very wicked and shout at clients.

Not all of the nurses are reliable and responsive to client because when I went to deliver, I has a cut and when one of the young nurses who conducted the delivery when out to call ne of the elderly nurses who sit at the table (a senior nurse) to come and check all she could say was that she is not ready to sting anything today. I had to lay down and wait for her before she came at her own convenient time to sting(sew) or me.

But others too are very reliable and very empathetic to clients because the last time I went to deliver I could not stand and my own after the baby came out and so one of the midwives came to hold and embrace me and told me to be patent and still and rest. some are really very supportive.

Even after delivery they use cotton to remove all the blood clots and during the process when you are trying to sit up, they tell you to please be patient with them so that they will clean you up to avoid after birth complication and access bleeding when you get home so I think they are empathetic to clients

Well for me the last time I went to deliver the nurses who sit at the table were very mean and not empathetic at all, because after I delivered and came out to the ward I was very tasty and they were selling water my money was only 10pesewas and I begged them to give me one pure water to rink so that when my people come I will get money to pay but they refused it was one lady who was sleeping next to me who gave me water to drink so to me they are mean

.

Oh yes for that aspect they are very timely to call to duty even if you are going to deliver, the young nurses run to meet you at the entrance to take your bag and give u a bed and start the checkup.

Yes the young nurses are very timely to call of duty unlike the elderly women who there previously the young nurses welcome you nicely and interact with you.

Yes there is communication during delivery when u get there and your time is due, they do not allow you to o out or walk around again they beg u lay on the bed and push and even when at a point you tell them that you want to ease yourself they tell you to do it on the bed and they will collect it and clean you up

and also immediately you deliver they ask you what you want and the they put the baby n you for five minutes that is baby to mother love.

**3.**Me it was Islamic hospital that I want to deliver and I was referred to regional hospital for CS but I lost the baby but between the two hospitals I did not see any difference in services they render .

No I have never been referred from one hospital to another I just go to the regional hospital when am in labour.

**4.**No for me am not satisfied with the overall maternal services of the regional hospital because they delay and waste our time a lot during the health talk when we are pregnant and go for the monthly checkups and even send our babies to weighing so I think they should always try to summarize what ever they want to talk about. Because am a teacher in one of the villages and anytime I have to seek permission from the headmaster and they end up delaying me all times which get the headmaster mad at me.

I also think the experienced nurses should educate the younger ones because the last time I went for one of the monthly checkups, one of the young nurses wrote that I should go and take a scan and that was around my seventh month on pregnancy but when I got to the table one of the elderly and experienced nurses told me that scan is only done during the ninth month so I had to go and come bad for them to rewrite the scan for the ninth month. So I think the young nurses are not very experienced because they calculate wrongly for clients which am not very happy about.

The processes are that when you get there they collect your health insurance card and maternity book. if you do not bring any of these they will not mind you because they cannot trace your history without it.

They will then give you a bed if you are not due and checkup regularly to see if are opening up.

if you deliver they the clean you up and give you some injections to stop too much bleeding and hold you back the ward to rest.

But it depends on the individual because for me I was induced and it took me so long to deliver but other women are so lucky because immediately the get there the baby comes out and they are free.

Yes, it is affordable to me because I paid ₵6 Ghana cedi’s. I was even lucky because most of the things I could not buy and the nurses told me to pay for was later dashed to me I did not pay before I was discharged.

Well to me it was affordable but it depends on the individual because some also come and they do not even have ₵1Ghana cedi on them. And also, the things that we buy the nurses should always try and take them as they are.

Yes, it is accessible to us especially with the plenty chips Zones around we will say it is very accessible to us. some of us run to the regional hospital all the time to avoid complications

Well they are doing well and we will reward them 90% especially the labour room nurses but for the elderly women who sit at the table in the main ward we will rate them 15% because all they do is to sit there and say annoying thing to women in pain. So, if here is any ward it should be for the midwives in the labour room because they have the human face and share your pain when you enter to deliver.

**Section IV: Challenges service users face in seeking quality healthcare during delivery**

1.The elderly nurses say things to clients that are not worth it. especially when they think you do not understand English language.

2.My main challenge was that the nurse where not content with the Dettol bought I had to buy another one which they were sill not okay with and because of that they spoke to me in a way that made the other women turned to look at me like I did something so bad which I did not like. they should know that all hands ae not equal and be content wit what each one has to bring.

I was also induced very early than the normal time and I went through so much pain from 6pm to down by a nurse who did not check me well.

After I delivered and came out to the ward I was very tasty and they were selling water my money was only 10pesewas and I begged them to give me one pure water to rink so that when my people come I will get money to pay but they refused it was one lady who was sleeping next to me who gave me water to drink so to me they are mean.

My challenge is that I do not know why I was operated on and when I asked they did not tell me.

**3.RECOMMENDATIONS**

The elderly nurses should learn to be patient and polite to clients.

They should also try to always to summarize whatever they have to tell us doing weighing and prenatal so that we can go about our normal activities.

Also, they should always try to keep the retain drugs for pregnant women at the place they do the checkups so that immediately after checkup you take your drugs and go home but not to refer us to go and join the long queue at the dispensary to take our drugs.

**Section II: Service users’ perception about what constitutes quality maternal healthcare**

Health care services without harassment, where the nurses don’t disturb you and take nothing from you during and after the care. When I was to deliver, I went with Dettol and parasol. The nurses used them and returned the remaining to me. The encouraged me to be coming from post-natal care services. They said anytime the baby is not feeling well I should bring her to the health care center.

**Section III: Appraisal of quality of maternal healthcare provided in WA municipality**

At the regional hospital, nurses harass people. I saw women who were being harassed when we went first. some of my colleagues who got pregnant whilst in school, they were insulting them and even refused to use the Dettol they bought and brought to the health facility. They told them that the Dettol was very old.

The facility in Kpongu here is better, the nurses are good and give us information about the weighing and Ante-natal care services. Even we are not able to come early, the nurses weight for us come and provide us the services to our children. The teach us the kind of food to feed our children.

But the rooms here are not spacious enough. We only have one bed, when there are two or more deliveries, both of them have to lay on it like that and it is a very small bed. Sometimes one has to lay on the floor for one to finish before. The nurses are enough, but only one is in-charged of deliveries. The midwife has given us her number so that anyone in labour can call her when she is not around. If she not around none of the nurses can perform deliveries. Some women either go to regional hospital to deliver when she is not around particularly in the night. The mid-wife used to sleep here but because of security issues, she goes to Wa to sleep.

The nurses are reliable, anytime you call her she comes around to attend to you. They don’t sleep here but they are responsive. For the midwifes when she even close and goes home and you call her to come and perform deliveries she comes back. I know the nurses have equipment for their work but I don’t know whether they are enough or not. I have never been referred to Wa before.

When you first arrive at the facility, they look at the antenatal care card and inspect the items they asked you to bring; the Dettol, the parasol, the rubber which they normally spread on the bed during the delivery, the pad and the clothes to cover the child after delivery. The services are affordable we don’t pay anything.

**Section IV: Challenges service users face in seeking quality healthcare during delivery**

Our facility is small and not spacious enough for many beds for deliveries. They are not enough midwives, just one we have here, when two women are on labour and one has complications the attention of the midwife is normally drawn completely to that person only leaving you behind to manage

**RECOMMENDATIONS**

If we get a more spacious facility with additional midwives it would have been helpful to us

**Section II: Service users’ perception about what constitutes quality maternal healthcare**

The constant checking they give every month and also the good diet recommendations and advice for instance, they tell you not to think too much, not to do hard work, not to get angry among others is quality maternal healthcare to me.

**Section III: Appraisal of quality of maternal healthcare provided in WA municipality**

The services they render are very good because they do not disturb or shout at pregnant women but rather take time to interact with us.

The facility has a spacious ward for delivery but just one bed in it which I think is not enough because which two or more women come at the same time to deliver only one will get a bed the others will not.

There are enough personnel here and also the tools and equipment for deliver are enough and functioning well. There are two (2) midwives here who conduct delivery and they are very reliable and responsive to clients because immediately you get here, they come to you and begin to interact with you till you deliver

The nurses are very empathetic to clients because they do not shout or treat clients badly they try to share you pain with you , they are patience with you in the pain you go through till you deliver

They are timely to call to duty because when you get here, and the nurses are doing any other thing aside attending to patients, they stop it immediately and attend to you.

Yes there is communication during delivery, the midwives give you encouragement to push so that the baby will come out and not die inside. No i haven never been referred from one hospital to another.

Yes am very satisfied with the overall maternal healthcare services received here because they care and are very patience with clients and also do their best to help you deliver safely and take your child in good health. The processes involved when I went to deliver were that took mu card and maternity book, checked my BP and items I brought(Dettol, parazon rags and other.) I was given a drip and asked to walk around until I felt like going to toilet and when the nurses checked me at that time they said I was due, I was then delivered, the placenta was also taken out and they clean me and my baby.

To me , I think the processes are okay. it is very affordable because the last time I came to deliver I paid just ₵2 Ghana cedi’s and so I think it affordable. it is accessible to me because it just by my house.

I will rate them one of the best because of the good work they do.

**Section IV: Challenges service users face in seeking quality healthcare during delivery**

The fact that I was cut before the baby came out was a challenge to me and other mothers.

Also the items that we mothers have to buy when we are to deliver is a challenge to us.

The beds are not enough even though the ward is spacious and that is a challenge to us because if u go there and some one is already there, you will have to be referred to WA regional hospital.

For me, my main challenge was for the baby to come out safely.

**RECOMMENDATIONS**

If they could add another bed to the one in the delivery room, it will help a lot.

Also there are times we buy some of the drugs because the health facility do not have. So we will be happy if they would provide all the drugs to us.

**Section II: Service users’ perception about what constitutes quality maternal healthcare**

It is how they treat at the facility. For me it is of good quality, the welcoming nature of the nurses at the facility here is really good. When I was pregnant and went they assisted me to deliver and they gave me some rest for sometime and discharged me. This facility is better than the regional hospital, because we are staying together and they have that humanity in them than the regional hospital nurses. If you go there and you don’t have patience all your sickness will varnish and you come home.

**Section III: Appraisal of quality of maternal healthcare provided in WA municipality**

Here, the facility is not spacious, they just took over a part of house and renovated it as health center so it is not spacious enough. When two women are in labour one either lay on the floor for the other to deliver. They are about 6 nurses, 3 permanent nurses and the 3 temporary nurses. When I went to deliver, two of them attended to me and the remaining one permanent nurse taking of the weighing of the children. I will say the nurses here are not enough because we the clients are many. For me I am not working there so I cannot tell whether they have all the tools or not. The conduct deliveries. They are reliable, I went there at dawn to wake them up to conduct the delivery and they attended to me. I delivered successfully from the CHP.

I am very satisfied because it is closer to us, I don’t go to the regional hospital to do weighing of my child, am really satisfied. All the wanted I sent; the Dettol, the parasol, the rubber and the cloths. Delivery is affordable no payment of money for deliveries. If some asks me to choose between this CHIP compound and the regional hospital, I will recommend the CHIP here rather.

**Section IV: Challenges service users face in seeking quality healthcare during delivery**

Our nurses are not many and the rooms are not spacious enough. Any time are they are conducting deliveries, they are always not enough nurses to cater for the weighing of the children.

**RECOMMENDATIONS**

If we get a more spacious facility with additional midwives, beds it would have been helpful to us. if we have more delivery rooms we will get more nurses to cater for the ANC and PNC services and attending to other sick persons. I faced this particularly challenge I went to deliver, not all the nurses sleep here, only the Zoom nurses sleep at the facility because of the limited number of rooms.

**Section II: Service users’ perception about what constitutes quality maternal healthcare**

They took care of me during the delivery, they gave me drugs and informed me of the kind of food to eat after birth. They said I should be eating fish, dawadawa, leaves, Kontonmiry stew and I used to eat a lot of that. It is really good for us.

**Section III: Appraisal of quality of maternal healthcare provided in WA municipality**

The rooms are not spacious enough. That I went to deliver, four of us gave birth that day. we were sitting down waiting for one to finish before the other because, the bed is only one. When one delivers then they start to supervise the next person. We were all lucky because whenever one birth is about coming out the one on the bed is done or almost close to completion. Only one midwife is available for deliveries and she work tirelessly all day. They are responsive and could prepare tea for us to drink after the delivery to gain more energy. They doing very well, during weighing, they more around the community and inform us of the dates and time. we cannot dispute that. I am really satisfied with their work. The beds are not enough but we are always lucky not to meet long queues during deliveries at the health center. We only pay Ghc 2.00 for light bill anytime we deliver at the facility. Everyone needs good and quality health care services after delivery. So if someone asks me to choose a health center for delivery, I will recommend this CHIP compound for the person.

**Section IV: Challenges service users face in seeking quality healthcare during delivery**

We only have one bed for delivery, when many women go for delivery then we have a lot of problems. The rooms are not also spacious enough. The nurses are not enough, if we get three midwives for deliveries that will be good. Also, we have not water source at the health care center where women can access water and bath after delivery. Mostly we have to contribute money every household every month to buy water for the facility for the women to bath after delivery.

**RECOMMENDATIONS**

If we get a more spacious facility with additional midwives, beds it would have been helpful to us. if we have more delivery rooms we will get more nurses to cater for the ANC and PNC services and attending to other sick persons. I faced this particularly challenge I went to deliver, not all the nurses sleep here, only the Zoom nurses sleep at the facility because of the limited number of rooms.
