# Supplementary material for: Is quality maternal healthcare all about successful childbirth? Views of mothers in the Wa Municipality, Ghana
Source: PLoS One. 2021 Sep 15;16(9):e0257401. doi: 10.1371/journal.pone.0257401 (PMC8443026; doi:10.1371/journal.pone.0257401)
Supplement: Attachment — Submitted filename: PLOS ONE review.docx [file pone.0257401.s002.docx]

Is quality maternal healthcare all about successful childbirth? views of mothers in the Wa Municipality, Ghana

Dear editor,

Thanks for inviting me as reviewer on the above titled manuscript for PLoS ONE.

First, the authors study a topic that is relevant for achieving the Substianable Development Goals 3 in one of the most underdeveloped regions of Ghana.

I will recommend that the manuscript is accepted with minor revisions. Below are my suggestions for the authors to consider in their revision.

1. The authors provide an overview of the maternal mortality ratio in the Wa municipality in the Upper West Region of Ghana. The authors state that the municipality is the worst performing in the region in terms of MMR. I will suggest that the authors provide the MMR statistics for the best performing municipality (or average of the region) to provide context to the readers of the situation of the municipality. Such a comparison will provide an additional justification for studying quality maternal healthcare in the municipality.
2. The authors should also provide a brief socioeconomic background of the Wa municipality (or the Upper West Region).
3. Does the choice of a convenient sampling procedure affect the results of the study? How do the authors address the limitations of the sampling method to reduce bias in the findings of the study?
